# Comparisons of Metabolic Measures to Predict T1D vs Detect a Preventive Treatment Effect in High-Risk Individuals

**DOI:** 10.1210/clinem/dgae048

**Published:** 2024-01-24

**Authors:** Emily K Sims, David Cuthbertson, Laura Jacobsen, Heba M Ismail, Brandon M Nathan, Kevan C Herold, Maria J Redondo, Jay Sosenko

**Affiliations:** Department of Pediatrics, Wells Center for Pediatric Research, Pediatric Endocrinology and Diabetology, and the Center for Diabetes and Metabolic Diseases, Indiana University School of Medicine, Indianapolis, IN 46202, USA; Department of Pediatrics, Pediatrics Epidemiology Center, Morsani College of Medicine, University of South Florida, Tampa, FL 33606, USA; Department of Pediatrics, University of Florida College of Medicine, Gainesville, FL 32610, USA; Department of Pediatrics, Wells Center for Pediatric Research, Pediatric Endocrinology and Diabetology, and the Center for Diabetes and Metabolic Diseases, Indiana University School of Medicine, Indianapolis, IN 46202, USA; Department of Pediatrics, University of Minnesota, Minneapolis, MN 55455, USA; Division of Diabetes and Endocrinology, Yale University, New Haven, CT 06520, USA; Departments of Immunobiology and Internal Medicine, Yale University, New Haven, CT 06520, USA; Texas Children's Hospital, Baylor College of Medicine, Houston, TX 77030, USA; Department of Medicine, Division of Diabetes, Metabolism, and Endocrinology, University of Miami, Miami, FL 33136, USA; Diabetes Research Institute, University of Miami, Miami, FL 33136, USA

**Keywords:** type 1 diabetes, C-peptide, glucose, prevention, prediction, insulin secretion, endpoint, trial

## Abstract

**Context:**

Metabolic measures are frequently used to predict type 1 diabetes (T1D) and to understand effects of disease-modifying therapies.

**Objective:**

Compare metabolic endpoints for their ability to detect preventive treatment effects and predict T1D.

**Methods:**

Six-month changes in metabolic endpoints were assessed for (1) detecting treatment effects by comparing placebo and treatment arms from the randomized controlled teplizumab prevention trial, a multicenter clinical trial investigating 14-day intravenous teplizumab infusion and (2) predicting T1D in the TrialNet Pathway to Prevention natural history study. For each metabolic measure, t-Values from t tests for detecting a treatment effect were compared with chi-square values from proportional hazards regression for predicting T1D. Participants in the teplizumab prevention trial and participants in the Pathway to Prevention study selected with the same inclusion criteria used for the teplizumab trial were studied.

**Results:**

Six-month changes in glucose-based endpoints predicted diabetes better than C-peptide–based endpoints, yet the latter were better at detecting a teplizumab effect. Combined measures of glucose and C-peptide were more balanced than measures of glucose alone or C-peptide alone for predicting diabetes and detecting a teplizumab effect.

**Conclusion:**

The capacity of a metabolic endpoint to detect a treatment effect does not necessarily correspond to its accuracy for predicting T1D. However, combined glucose and C-peptide endpoints appear to be effective for both predicting diabetes and detecting a response to immunotherapy. These findings suggest that combined glucose and C-peptide endpoints should be incorporated into the design of future T1D prevention trials.

The recent clinical trial showing that teplizumab (anti-CD3) can delay the diagnosis of type 1 diabetes (T1D) for 32.5 months has reinvigorated the quest to prevent the disorder (1, 2). However, even for the high-risk participants with Stage 2 T1D in the teplizumab trial, determination of treatment efficacy required years of follow-up before the necessary number of participants developed the primary endpoint of Stage 3 (clinical) T1D.

Given the likelihood of an increasing number of prevention trials, there is a practical need to shorten these trials. Post hoc metabolic analyses of the teplizumab (3) and oral insulin type 1 diabetes prevention trials (4) suggest that this is possible. Metabolic endpoints detected a treatment effect at 3 and 6 months of follow-up in the teplizumab trial, and at 1 year of follow-up in the oral insulin trials.

The value of metabolic endpoints is a function of their capacity to both predict type 1 diabetes and to detect a treatment effect. However, assessments of this combined capacity for prediction and detection have not been performed. Thus, we analyzed metabolic endpoints for their ability to both (1) detect treatment effects in the teplizumab prevention trial, and (2) accurately predict type 1 diabetes in the TrialNet Pathway to Prevention Study (TNPTP), in which autoantibody-positive individuals are followed observationally. These assessments should help to determine which metabolic endpoints would be most useful for prevention trials.

## Materials and Methods

### Studies Analyzed

The randomized controlled TN10 TrialNet teplizumab prevention trial (NCT01030861), which was completed in 2019, tested the impact of a 14-day course of teplizumab compared with placebo on time to type 1 diabetes in individuals ≥8 years old with ≥2 autoantibodies and dysglycemia, and a family history of type 1 diabetes (1). The TNPTP is an ongoing longitudinal observational study in which individuals with a family history of type 1 diabetes are screened for islet autoantibodies (5). Those positive for multiple islet autoantibodies are followed at 6- or 12-month intervals with 2-hour oral glucose tolerance tests (OGTTs) for diagnostic surveillance. To ensure consistency and independence among the groups studied, only TNPTP participants who would have met the criteria for the teplizumab trial but did not participate in it or other intervention trials were included (n = 174, 93/174 developed stage 3 type 1 diabetes during follow-up). Informed consent and assent (when appropriate) were obtained from all participants, with investigations carried out in accordance with the principles of the Declaration of Helsinki as revised in 2008. For the teplizumab prevention trial, ethics approval was obtained by institutional review boards at each study site. For TNPTP, ethics approval is maintained at a central institutional review board.

OGTT glucose and C-peptide data measured as part of both original studies were obtained from the TrialNet Coordinating Center (1, 5). C-peptide measurements were performed using the TOSOH immunoassay (Tosoh Biosciences Cat# 025283, RRID:AB_3083552). OGTT glucose and C-peptide assays and metabolic endpoint calculations included area under the curve (AUC) C-peptide, AUC glucose, AUC ratio (AUC C-peptide/AUC glucose × 1000), 30-0 minute C-peptide, Index60 (6-month within quadrant endpoint [WQE]), and 6-month ordinal directional endpoint (ODE). Index 60 was calculated as (0.36953 × (log fasting C-peptide [ng/mL]) + 0.0165 × glucose 60 (mg/dL) − 0.3644 × C-peptide 60 (ng/mL)) (6). Since both WQE and ODE have previously been explained in detail elsewhere (2, 3, 6, 7), they will briefly be explained here. WQE and ODE both quantify differences between groups in changes of directional movement of glucose and C-peptide response curves (GCRCs) from OGTTs on a 2-dimensional grid, with glucose values as the ordinate and C-peptide values as the abscissa. They are used to calculate differences in angles between vectors of GCRC movement over a 6-month period on the grid. For instance, in the post hoc analysis of the teplizumab trial (3), vector angles of GCRC movement from baseline to 6 months were compared between the placebo and treatment groups. The centroid (central point) of the baseline GCRC and 6-month GCRC were connected to form the vectors. Trigonometric calculations were then utilized to compare the vector angle between the placebo and treatment groups. WQE and ODE were developed with the same objectives, but they represent 2 approaches for estimating differences between vector angles. They are unique endpoints since they are the only measures that provide quantitative assessments of differences in directional changes of OGTTs (as indicated by GCRCs) between groups. The post hoc teplizumab report (3) provides more detailed information regarding WQE and ODE.

### Data Analysis

Changes (ie, arithmetic differences) in metabolic endpoints from randomization were compared for their capacity to detect a treatment effect of teplizumab in the 68 participants with available data at the 6-month timepoint (n = 44 for teplizumab treated and n = 24 for placebo treated), using t-tests and analyses of covariance. Results were also adjusted for baseline age, body mass index (BMI), and parameter of interest except for WQE and ODE, which were adjusted for baseline age and BMI, AUC glucose and AUC C-peptide. Strength of association between type 1 diabetes occurrence and 6-month changes in metabolic endpoints was tested using chi-square values from Cox regression (run both unadjusted and adjusted as above). To address differences in follow-up for those who did not develop type 1 diabetes, participants were censored as of their last OGTT. Kaplan–Meier curves were also generated for TNPTP participants above and below the median for each metabolic endpoint and compared using log-rank tests. Chi-square values for predicting type 1 diabetes were plotted against t-values for detecting a treatment effect for each metabolic measure to assess their capacities relative to each other. SAS 9.4 was used for analyses; 2-sided *P* ≤ .05 was considered to be statistically significant.

## Results

Baseline characteristics of the placebo (n = 24) and treatment (n = 44) groups from the teplizumab trial, and individuals from the TNPTP cohort meeting the teplizumab trial inclusion criteria (1) (n = 174), are shown in Table 1.

**Table 1.** Participant characteristics at baseline

	Teplizumab placebo group (n = 24)	Teplizumab treatment group (n = 44)	TNPTP (n = 174)
Sex: female, n (%)	10 (41.7)	19 (43.2)	82 (47.1)
**Race, n (%)**			
Asian	1(4.2)	0 (0.0)	4 (2.3)
Black or African American	0 (0.00)	0 (0.0)	4 (2.3)
White	23 (95.8)	44 (100)	147 (86.0)
>1 race	0 (0.00)	0 (0.0)	2 (1.2)
Unknown or not reported	0 (0.00)	0 (0.0)	12 (7.0)
**Ethnicity, n (%)**			
Hispanic	1 (4.2)	1 (2.27)	16 (9.3)
Unknown	1 (4.2)	0 (0.0)	10 (5.8)
Age (years)	16.0 (9.4); (8.5-43.0)	19.2 (11.9); (8.5-49.4)	17.0 (10.8); (8.1-46.3)
BMI (kg/m^2^)	21.7 (4.5); (16.1- 34.6)	22.0 (6.4); (14.7-43.8)	22.4(6.6); (13.9-53.7)
AUC glucose	158.0 (22.5)	164.9 (23.5)	163.2 (19.8)
HbA1c (%)	5.2 (0.3)	5.2 (0.4)	5.3 (0.4)
AUC C-peptide	6.1 (2.5)	6.1 (2.6)	6.5 (2.6)
30-0 minute C-peptide	3.5 (1.9)	3.1 (1.9)	3.7 (2.2)
AUC Ratio	3.8 (1.5)	3.7 (1.5)	4.0 (1.6)
Index60	0.6 (0.9)	0.8 (1.0)	0.6 (1.1)
Follow up time (years)	2.9 (2.1); (0.3-7.0)	3.4 (2.1); (0.0-9.1)	2.8 (2.9); (0.2-15.1)

Sex, race, and ethnicity data were self-identified by participants or their parents; data shown as mean (SD); (min-max) unless otherwise noted.

Abbreviations: AUC, area under the curve; BMI, body mass index.

**Figure 1.**
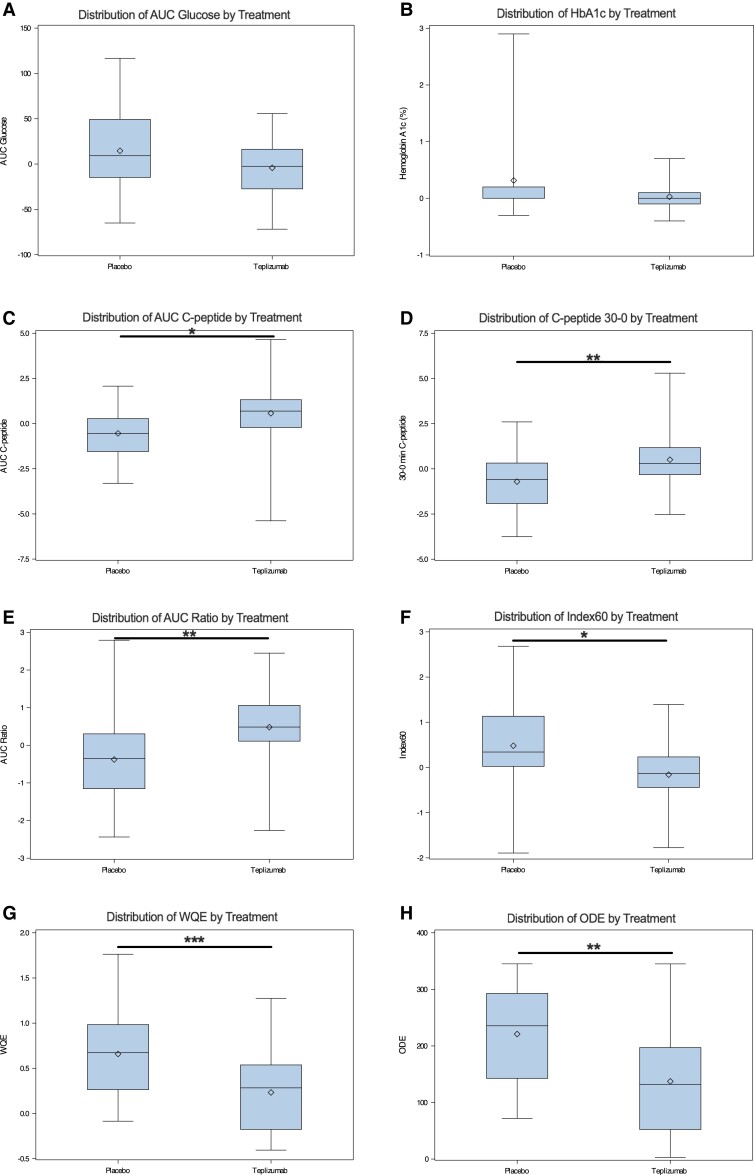
The 0- to 6-month changes in metabolic measures in teplizumab prevention study participants. Changes are presented stratified by treatment group. Statistical comparisons are presented for comparisons adjusted for baseline age, BMI, and parameter of interest except for WQE and ODE, which were adjusted for baseline age, BMI, glucose AUC, and C-peptide AUC. Skeletal boxplots display: minimum and maximum values (whiskers), 25th and 75th percentiles (top and bottom of box), median (center line), and mean (asterisk within box). *P < .05; **P < .01; ***P < .001. n = 24 for placebo-treated group and n = 44 for teplizumab-treated, except for HbA1c, where n = 14 and n = 30 respectively.

Table 2 includes baseline and 6-month values, as well as comparisons of 0- to 6-month changes with associated t-values for metabolic endpoints in the placebo and teplizumab groups. Box and whisker plots for 0- to 6-month changes are shown in Fig. 1. Comparisons between the placebo and teplizumab groups of the 6-month changes in measures of C-peptide, either alone or in combination with glucose, showed a beneficial treatment effect (*P* < .05 for all), with and without adjustments for age, BMI, and baseline value. The difference was greatest for WQE (*P* < .001 unadjusted and adjusted). Measures that only utilized glucose did not show a significant treatment effect (*P* > .05).

**Table 2.** Detection of a 6-month treatment effect in the teplizumab prevention trial

	Placebo (n = 24):			Teplizumab (n = 44):				
Parameter	Baseline	6 months	6-month change	Baseline	6 months	6 month change	t-value unadjusted/adjusted	*P* value unadjusted/adjusted
AUC Glucose	158.00 (22.53)	172.59 (51)	14.585 (48.01)	164.91 (23.50)	160.70 (24.22)	−4.209 (29.88)	3.96/−1.61	.051/.112
Hemoglobin A1c (%)	5.18 (0.34)	5.45 (0.77)	0.314 (0.83)	5.19 (0.37)	5.22 (0.37)	0.028 (0.25)	3.00/−1.63	.091/.112
AUC C-peptide	6.12 (2.49)	5.58 (2.73)	−0.542 (1.41)	6.06 (2.61)	6.62 (2.42)	0.569 (1.77)	7.04/2.51	.010/.015
30-0 minutes C-peptide	3.53 (1.94)	2.82 (1.86)	−0.709 (1.54)	3.14 (1.88)	3.64 (1.67)	0.507 (1.53)	9.71/2.77	.003/.007
AUC ratio	3.83 (1.4)	3.45 (1.77)	−0.379 (1.17)	3.68 (1.52)	4.16 (1.4)	0.481 (0.98)	10.45/2.90	.002/.005
Index60	0.56 (0.92)	1.04 (1.51)	0.481 (1.10)	0.80 (1.00)	0.64 (0.94)	−0.159 (0.77)	7.85/−2.37	.007/.021
WQE	n/a	0.66 (0.47)	n/a	n/a	0.23 (0.46)	n/a	13.19/−3.57	<.001/<.001
ODE	n/a	221.0 (87.0)	n/a	n/a	137.4 (94.0)	n/a	12.92/−3.28	<.001/.002

Data shown as mean (SD). Data for HbA1c was only available for 14 placebo-treated participants and 30 teplizumab treated participants. Values adjusted for baseline age, BMI, and parameter of interest except for WQE and ODE, which were adjusted for baseline age, BMI, AUC glucose, and C-peptide. AUC ratio reflects the ratio of AUC C-peptide/AUC glucose × 1000. There is no baseline measure for WQE and ODE per se, since by definition it is a 6-month measure. However, it is still built upon percent change from baseline.

Abbreviations: AUC, area under the curve; BMI, body mass index; ODE, ordinal directional endpoint; WQE, within quadrant endpoint.

**Figure 2.**
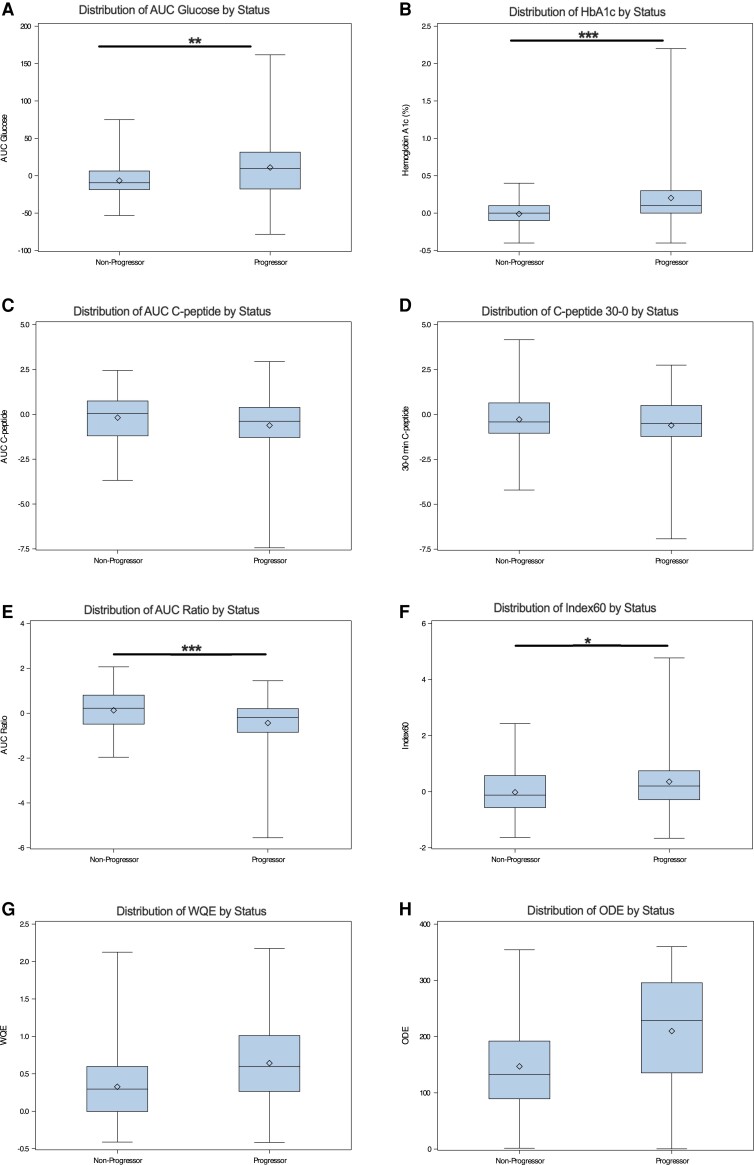
The 0- to 6-month changes in metabolic measures in PTP participants meeting teplizumab prevention study entry criteria. Changes are presented stratified by progression status. Statistical comparisons are presented for comparisons adjusted for baseline age, BMI, and parameter of interest except for WQE and ODE, which were adjusted for baseline age, BMI, glucose AUC, and C-peptide AUC. Skeletal boxplots display minimum and maximum values (whiskers), 25th and 75th percentiles (top and bottom of box), median (center line), and mean (diamond within box). *P < .05; **P < .01; ***P < .001. n = 81 for nonprogressors and 93 for progressors, except for HbA1c, where n = 76 for nonprogressors and 86 for progressors.

As shown in Table 3, we also examined the prediction of Stage 3 type 1 diabetes in TNPTP participants with proportional hazards regression. Box and whisker plots for 0- to 6-month changes are shown in Fig. 2. Without adjustment, according to the chi-square values, the 6-month change in AUC glucose was the strongest predictor (*P* < .001) of diabetes progression, although changes in HbA1c, Index60, AUC ratio, WQE, and ODE were also highly predictive (*P* < .001). With adjustment, diabetes progression was significantly associated with all variables. Kaplan–Meier curves were also generated for participants above or below median values of each measure for the entire group (Figs. S1-S8 (8)) and were consistent with findings in Table 3. Additionally, although teplizumab study participants composed a much smaller sample size, we performed Cox regression for each measure for those individuals, where we also observed similar associations with type 1 diabetes progression (Table S1 (8)).

**Table 3.** Associations of type 1 diabetes prediction with 6-month changes in metabolic measures in TNPTP participants meeting entry criteria for teplizumab prevention trial

Parameter	Hazard ratio (95% CI)	Chi-square (unadjusted/adjusted)	*P* value (unadjusted/adjusted)
AUC glucose	1.022 (1.015-1.028)	34.6/43.5	<.001/<.001
HbA1c (%)	4.128 (2.387-7.138)	29.7/25.7	<.001/<.001
AUC C-peptide	0.750 (0.622-0.905)	3.2/9.0	.075/.003
30-0 minutes C-peptide	0.719 (0.601-0.860)	3.7/13.0	.054/<.001
AUC ratio	0.463 (0.362-0.592)	23.0/37.6	<.001/<.001
Index60	2.079 (1.634-2.644)	17.0/35.5	<.001/<.001
WQE	1.986 (1.427-2.765)	17.5/16.5	<.001/<.001
ODE	1.005 (1.003-1.007)	24.3/19.9	<.001/<.001

N = 174, except for data for HbA1c which was only available for 164 individuals. Adjusted values were adjusted for baseline age, BMI, and baseline parameter except for WQE and ODE which were adjusted for baseline age, BMI, AUC glucose and C-peptide. AUC ratio reflects the ratio of AUC C-peptide/AUC glucose × 1000.

Abbreviations: AUC, area under the curve; BMI, body mass index; ODE, ordinal directional endpoint; WQE, within quadrant endpoint.

**Figure 3.**
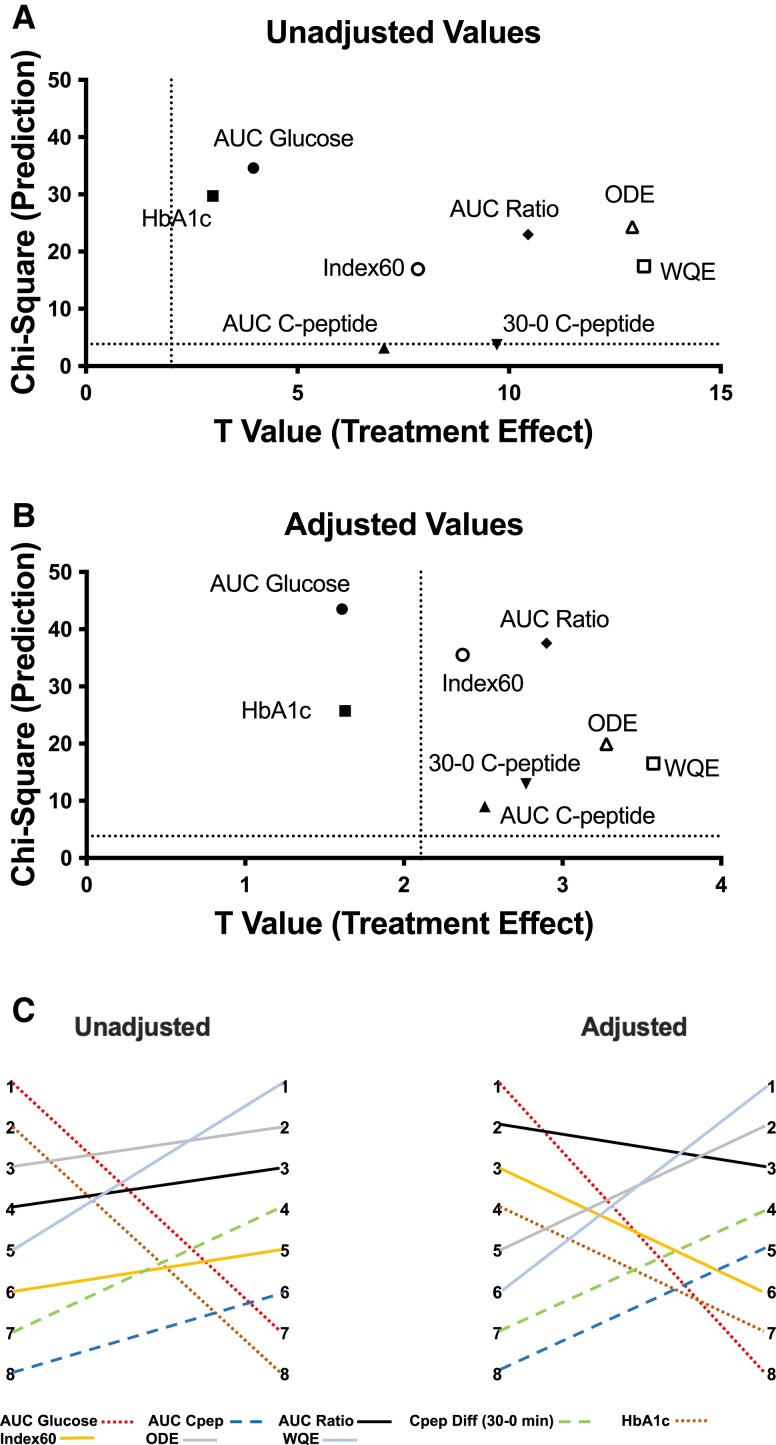
Discrepancy in ability to detect a treatment effect in the teplizumab trial vs associations with type 1 diabetes progression in the TNPTP study. For change in each variable over a 6-month period, absolute t-values generated by comparisons of teplizumab vs placebo treatment groups (x axis) vs absolute chi-square values generated by Cox regression analyses for relationship with time to diabetes (y axis) are plotted. (A) Unadjusted values. (B) Values adjusted for baseline age, BMI, and variable of interest, except for ODE and WQE, which were adjusted for baseline AUC glucose and AUC C-peptide along with baseline age and BMI. Dashed lines represent a t-value value and Chi-Square value that reflect a *P* value of .05, such that values above or to the right of these thresholds meet statistical significance. (C) Rankings of change in each metabolic variable over a 6-month period for predicting type 1 diabetes (based upon chi-square values) and for detecting a treatment effect (based on t-values) are shown for unadjusted and adjusted data shown in A and B. Color coding is used to indicate each metabolic measure as described in the legend. Dotted lines represent the 2 “glucose only” measures. Dashed lines represent the 2 “C-peptide only” measures. Solid lines represent the 4 combined measures. n = 68 for t-values except for HbA1c, where n = 44. n = 174 for chi-square values, except for A1c, where n = 167.

Figure 3A and 3B graphically shows metabolic endpoint positions, with chi-square values for diabetes prediction on the y-axis (higher y values indicating better prediction) and with t-values for treatment effect detection on the x-axis (higher x values indicating better detection of a treatment effect) with and without adjustments for age, BMI, and the baseline measure. Endpoint positions varied substantially. Glucose-based measures were in the upper left region, indicating relatively better prediction of type 1 diabetes vs treatment effect detection. In contrast, C-peptide measures were in the lower central region, indicating relatively better detection than prediction. Index60 was in the upper central region, while ODE and WQE were shifted further to the right, indicating a greater ability to detect a teplizumab treatment effect. Positions of the combined glucose and C-peptide variables indicated better detection of a treatment effect than the glucose measures, and better type 1 diabetes prediction than the C-peptide measures.

Figure 3C further demonstrates the dissociation between the prediction of type 1 diabetes and the detection of a treatment effect. For both unadjusted and adjusted values, 2 columns are shown with the column on the left indicating the ranking of measures from 1 to 8 (1 = best, 8 = worst) for predicting type 1 diabetes and the column on the right indicating the ranking of measures from 1 to 8 (1 = best, 8 = worst) for detecting a treatment effect. Without adjustments, AUC glucose went from a rank of 1 for predicting type 1 diabetes to a rank of 7 for detecting a teplizumab effect. With adjustments, AUC glucose went from 1 for prediction to 8 for detection. In contrast, combined glucose and C-peptide endpoints, WQE and ODE, both with a midrange rank for prediction, were the 2 best measures at detecting treatment effects in the unadjusted and adjusted analyses.

## Discussion

This study was prompted by recent post hoc analyses of the teplizumab and oral insulin prevention trials (3, 4). Furthermore, the abatacept prevention study, although not meeting its primary endpoint, showed that active drug improved C-peptide AUC after 12 months of treatment (9). These results suggest that metabolic endpoints can provide valuable additional information, including the timing and nature of a treatment effect, or as an early or more sensitive readout of a treatment effect. Such findings could warrant the inclusion of metabolic measures as secondary endpoints in future prevention trial protocols.

If these measures are to be included in such trials, it is important to consider whether they should be viewed as surrogate endpoints. An ideal surrogate endpoint would not only detect an earlier treatment effect, but also be highly predictive of a diagnostic endpoint. We thus chose to study metabolic measures for their ability to predict Stage 3 type 1 diabetes and to detect a treatment effect. Our data strongly suggest that the ability of metabolic endpoints to predict type 1 diabetes does not necessarily correlate with their ability to detect a treatment effect.

A key finding was that among the changes in the metabolic measures from baseline to 6 months, the change in AUC glucose was a strong predictor of type 1 diabetes, yet it was not sensitive for detecting a disease-modifying teplizumab treatment effect. Since the thresholds used for a diagnosis of type 1 diabetes in the TNPTP are glucose based (10) (as in the clinical realm), glucose measures might be expected to be better predictors than either C-peptide measures or combined measures.

The fact that AUC glucose is a good predictor would not necessarily translate to AUC glucose being a good endpoint for detecting a treatment effect, however. We have previously shown that in normal, abnormal, and diagnostic ranges of glycemia, Index60, which combines OGTT glucose and C-peptide measures, is more strongly associated with features of type 1 diabetes (younger age, higher percentage of islet autoantibodies, lower BMI, high risk HLA genotypes) than are glucose measures (11, 12). These findings suggest that there is heterogeneity of glucose levels among autoantibody positive individuals due to other contributing factors besides β-cell compromise, such as insulin resistance. Since the effect of most potential preventive treatments (like teplizumab) are based on mechanisms targeting the autoimmune pathophysiology for β-cell loss, their effects might be better detected by metabolic measures more specific for pathologic features of type 1 diabetes. Other potential contributors to glucose levels would be less likely to respond to immunotherapy and thus obscure a treatment effect.

The suboptimal prediction of combined measures does not necessarily diminish the potential value of using them as endpoints for prevention trials. Since those measures appear to be more specific for mechanistically based treatments, they provide additional information beyond whether or not the diagnosis is delayed by a treatment. It is quite possible that a treatment could be negative for the glucose-based time to diagnosis endpoint, yet be positive for a combined endpoint that would be more sensitive to the mechanistic effects of a treatment.

We chose to focus on a 6-month timepoint for practical reasons: OGTTs are usually performed at 6-month intervals in the TNPTP, and in TN10 a substantial percentage of placebo-treated participants were removed from OGTT monitoring due to the development of diabetes after 6 months. If available in future studies, similar comparisons over shorter or longer periods of follow-up would be informative.

There were strengths and limitations in our study. A strength was the use of the extensive TrialNet database of at-risk individuals to predict diabetes and detect a treatment effect. There have been no prior studies of the consistency between the prediction and detection of preventive treatment effects for type 1 diabetes. A limitation was the potential for bias in a post hoc analysis. The prediction of type 1 diabetes after 6 months of follow-up was limited to individuals in the TNPTP cohort who were at high risk for type 1 diabetes, similar to risks of those selected for the teplizumab trial. Thus, the findings are not necessarily generalizable to other treatments and to other study populations. For instance, in prior studies we have observed that Index60 is a better baseline predictor of type 1 diabetes than glucose measures among those at lower risk (9, 10). A limitation in this study, and type 1 diabetes research studies in general, is a lack of racial and ethnic diversity (13, 14).

In conclusion, the findings revealed a seeming paradox in which glucose appeared to perform more effectively for predicting type 1 diabetes, but less effectively for detecting a treatment effect of teplizumab. These findings provide information to guide the design of future type 1 diabetes prevention trials. Furthermore, they challenge the current paradigm that a diagnosis of type 1 diabetes based on glucose alone suffices as the only endpoint of value in prevention trials. It appears that combined glucose and C-peptide measures should also be included as primary or at least secondary endpoints.

## Data Availability

Data are available upon reasonable request from the authors.

## Abbreviations

AUC: area under the curve
BMI: body mass index
GCRC: glucose and C-peptide response curve
ODE: ordinal directional endpoint
OGTT: oral glucose tolerance test
TNPTP: TrialNet Pathway to Prevention Study
T1D: type 1 diabetes
WQE: within quadrant endpoint
